# Supplementary material for: Coronary Microvascular Dysfunction in Takotsubo Syndrome Assessed by Angiography-Derived Index of Microcirculatory Resistance: A Pressure-Wire-Free Tool
Source: J Clin Med. 2021 Sep 23;10(19):4331. doi: 10.3390/jcm10194331 (PMC8509411; doi:10.3390/jcm10194331)
Supplement: Supplementary file 1 [file jcm-10-04331-s001.zip › jcm-1344536-supplementary.pdf]

Table S1. NH-IMRangio by each artery in different patterns of wall motion abnormalities

Pattern of wall motion abnormality:	n:	NH-IMRangio	
**LEFT ANTERIOR DESCENDING** (n: 166 patients)			
**Pattern of wall motion abnormality:**	**n:**	**NH-IMRangio**	**p: 0.0152**
- Apical limited	83	41.38 (28.71-51.18)	
- Midventricular & apical	42	56.98 (40.58-70.73)	
- Midventricular limited	18	35.34 (26.21-59.93)	
- Basal limited	10	41.19 (26.83-49.79)	
- Midventricular & basal	5	73.11 (69.34-76.91)	
- Others	8	50.20 (41.19-55.48)	
**CIRCUMFLEX LEFT ARTERY** (n: 156 patients)			
**Pattern of wall motion abnormality:**	**n:**	**NH-IMRangio**	**p: 0.1869**
- Apical limited	78	29.47 (21.29-47.54)	
- Midventricular & apical	41	41.24 (23.75-53.08)	
- Midventricular limited	17	30.71 (25.17-35.47)	
- Basal limited	8	26.70 (18.90-40.50)	
- Midventricular & basal	5	63.55 (55.31-71.30)	
- Others	7	40.18 (24.80-58.46)	
**RIGHT CORONARY ARTERY** (n: 141 patients)			
**Pattern of wall motion abnormality:**	**n:**	**NH-IMRangio**	**p: 0.0189**
- Apical limited	66	33.41 (19.96-44.64)	
- Midventricular & apical	37	44.61 (31.58-59.31)	
- Midventricular limited	17	38.00 (26.57-52.34)	
- Basal limited	10	27.96 (22.83-41.44)	
- Midventricular & basal	5	67.83 (62.90-73.13)	
- Others	6	33.36 (30.02-43.72)	

Variables are expressed as median (IQR), NH-IMRangio: non-hyperemic angiography-derived index of microcirculatory.

Table S2 – Differences in patients based on the number of arteries with CMD (n: 166 patients)

	One artery affected (n: 17)	Two arteries affected (n: 67)	Three arteries affected (n: 82)	p
Age (years)	81.3 (70.1-82.7)	72.3 (65.0-80.9)	75.6 (64.3-83.0)	0.1348
Female gender	23.5	19.4	86.6	0.462
Prior physical stressful trigger	52.9	35.8	36.6	0.450
Prior emotional stressful trigger	5.9	20.9	20.7	0.473
Dyskinesias location:				0.133
- Apical limited	64.7	50.2	42.7	0.063
- Mid-ventricular + Apical	11.8	22.9	35.4	0.020
- Mid-ventricular limited	17.7	10.5	12.2	0.717
- Basal limited	5.9	7.5	4.9	0.804
- Mid-ventricular + Basal	0	0	2.4	0.355
- Other	0	9.0	2.4	0.112
SBP (mmHg)	135.5 (117-143.5)	130 (115-150)	128 (115-143)	0.8343
Killip class at admission:				0.195
- I	41.2	67.2	68.3	0.092
- II	29.4	10.5	17.1	0.140
- III	11.8	10.5	9.8	0.967
- IV	17.7	11.9	4.9	0.139
Heart rate (bpm)	90.5 (80-115)	86.5 (75-100)	81 (73-93)	0.0844

Atrial fibrilation	5.9	26.0	7.3	0.939
End-diastolic LV pressure (mmHg)	17 (14-19)	18 (12-25)	18 (12.5-24.5)	0.8588
LVEF (%)	51 (41-62)	44 (35-58)	44 (38-52)	0.1635
pH	7.31 (7.25-7.36)	7.33 (7.26-7.4)	7.42 (7.34-7.44)	0.3207
Lactate (mmol/L)	5.2 (3-7.3)	2.5 (1.1-3.5)	2.3 (0.8-3.1)	0.3675
Hemoglobin (g/L)	125.5 (109-132)	130 (115-143)	130 (117-140)	0.2596
NT-proBNP (pg/mL)	2094 (200-4550)	2696 (1588-4166)	3650 (1924-7400)	0.0031
hs-cTnT (ng/L)	346 (61-558.5)	222 (94-735)	260 (64-643)	0.8283

Continuous variables are expressed as median (IQR) and categorical data as %. SBP: systolic blood pressure; LVEF: left ventricle ejection fraction; hs-cTnT: high-sensitive cardiac Troponin T
